# Does California’s Low Carbon Fuel Standards reduce carbon dioxide emissions?

**DOI:** 10.1371/journal.pone.0203167

**Published:** 2018-09-17

**Authors:** Samir Huseynov, Marco A. Palma

**Affiliations:** Department of Agricultural Economics, Texas A&M University, College Station, Texas, United States of America; Universitat Jaume I, SPAIN

## Abstract

The Low Carbon Fuel Standards (LCFS) represents a new policy approach designed to reduce carbon dioxide emissions by applying standards to all stages of motor fuel production. We use the synthetic control and difference-in-differences econometric methods, and Lasso machine learning to analyze the effect of the LCFS on emissions in California’s transportation sector. The three different techniques provide robust evidence that the LCFS reduced carbon dioxide emissions in California’s transportation sector by around 10%. Furthermore, our calculations show that improved air quality, due to the application of the LCFS, may have benefited California in the magnitude of hundreds of millions of dollars through an increase in worker’s productivity.

## Introduction

California’s transportation sector is the largest carbon dioxide producer in the state, accounting for nearly 37% of total emissions [1, 2]. On January 18, 2007, California launched the Low Carbon Fuel Standards (LCFS) program as an attempt to reduce the carbon dioxide intensity of motor fuels for on-road light-duty vehicles [3]. Light-duty vehicles accounted for 70% of transportation emissions in 2014. Although six additional states, including Washington, Arizona, New Mexico, Minnesota, Illinois, and Oregon intended to adopt similar policies, none of them succeeded in implementing similar standards [4]. In this article, we analyze whether the LCFS has reduced carbon dioxide emissions in California’s transportation sector. To assess causality, we apply different identification strategies using econometric techniques and machine learning. We start our analysis using the synthetic control method (SCM) [5–7]. Furthermore, we conduct two additional robustness check analyses using Difference-In-Differences (DID) and Lasso [8, 9], which is a machine learning approach. All three methods provide robust evidence that the LCFS significantly reduced the *CO*_2_ inventory in California. The magnitude of the *CO*_2_ emissions reduction for the transportation sector is around 10%.

There are three motivations for our study. First, some studies have assessed the outcomes of the LCFS; however, they mainly focus on the carbon dioxide intensity of all motor fuels in California as the outcome variable. For example, [10] find that since it’s implementation, the LCFS has decreased the carbon dioxide intensity of alternative fuels in California by around 15%. However, since the primary goal of the LCFS is to reduce carbon dioxide emissions in the transportation sector, we focus directly on this sector and quantify changes in the carbon dioxide inventory due to the LCFS.

Second, since the day it was implemented, the LCFS has been challenged by several stakeholders due to its life-cycle accounting method [11]. The life-cycle accounting assigns different pollution scores to different ethanol producers. In fact, the LCFS assigns higher pollution scores to Midwest ethanol producers since they mostly use coal in the production of ethanol. Out-of-state producers claim that this procedure harms their businesses by excluding their products from the California market. The U.S. 9^*th*^ Circuit Court ruled that the LCFS would reduce carbon dioxide emissions and benefit California residents stating that “California should be encouraged to continue and to expand its efforts” (see http://articles.latimes.com/2013/sep/18/business/la-fi-carbon-footprint-20130919). In short, the publicity with high expectations about the effectiveness of the LCFS in reducing *CO*_2_ emissions was the main reason for its legal support. From this perspective, the initial outcomes of the LCFS constitute a key factor in the determination of whether this policy has met expectations.

Third, California pioneers several environmental initiatives and the outcome of this policy will provide useful insights for federal policy and for other states considering similar strategies. The performance of the LCFS in California has high information value to other states, and if successful, it can generate a new wave of federal policy [12]. Therefore, studying the performance and consequences of the California LCFS is important for many interest groups. By applying a clear identification strategy, this paper estimates the outcomes of the LCFS, and it can serve as a reference point for the implementation of federal policy.

## Main regulatory elements of the LCFS

The LCFS constitutes a bundle of standards that aim to incentivize technological advancements to generate low-carbon fuels. It targets all transportation fuels for on-road vehicles [3]. The LCFS functions by restricting the carbon dioxide intensity (CI) of fuels offered by regulated parties and the goal is to achieve a 10% reduction by 2020 compared to the 2010 baseline [3, 13]. Regulated parties include all entities that either produce or import motor fuels for consumption in California [13]. The targeted compliance level is back-loaded, since the compliance threshold for the CI reduction starts with low values, but it increases rapidly in subsequent years [3]. [3] point out that, the back-loaded nature of the policy enables the private sector to invest in technological improvements in the early stages and benefit later stages when the compliance levels are higher.

If a supplier of motor fuels exceeds the compliance target, then the excess compliance is credited to the supplier’s account. Credits can be banked and used in subsequent periods to cover deficits, or the supplier can sell credits in the market. Alternatively, the supplier can also pay fines for non-compliance.

The production of low-carbon fuels also generates carbon dioxide emissions, it is hard to achieve one-to-one displacement of regular fuels by low-carbon fuels [14]. Thus, *E*_*displaced*, *t*_ (MJ/year) can be expressed as:

$$\begin{array}{c} E_{displaced,t}=E_{low\;carbon,t}-E_{CO2\;emissions,t} \end{array}$$ (1)

where *E*_*low carbon*,*t*_ is the supplied amount of low-carbon fuels and *E*_*CO*2*emissions*,*t*_ is the regular fuel equivalent of carbon dioxide emissions generated during the production of low-carbon fuels.

In order to prevent crude oil “shuffling”, refiners get the same CI rating for crude oil imported from different places [3, 15]. The main feature of the LCFS is considering life-cycle in the production of motor fuels. Under the Lifecycle model that estimates CI of fuel pathways, fuels are assigned different CI scores depending on fuel types, feedstock type and farming methods, transportation means, production processes, and by-products [3].

The LCFS constitutes one of the first attempts to bring the life-cycle carbon dioxide emission concept into the policy framework. Thus, the LCFS can serve as an alternative policy tool. Since the LCFS targets all steps in the production process, it stimulates more innovation than carbon dioxide taxes and cap and trade programs [16, 17]. The LCFS can also be compared to other policy standards, such as the Renewable Fuel Standards (RFS) adopted by the federal government in 2005. The RFS requires that 36 billion gallons of biofuels be sold annually by 2022 [16]. However, under the RFS, if the regulatory agency approves a specific biofuel pathway, then producers are left with little incentives to design more efficient ways of compliance, including waste biomass [16, 18].

The relevant economic literature has intensively studied Pigouvian taxes and Cap and Trade based policies and their consequences. Unfortunately, the LCFS type policies have not received enough scrutiny by economists since they are relatively new. From this perspective studying California’s recent law and its consequences provides a valuable opportunity to analyze the performance of LCFS type policies.

## Identification strategy

It is difficult to single out the effect of a particular policy from other related policies. The situation becomes even more cumbersome when various state and federal laws overlap [12]. Since the LCFS targets carbon dioxide emissions in the transportation sector, in this section we focus on federal and state level regulations that aim at decreasing pollution caused by on-road vehicles (see Table 1).

**Table 1:** Different identification strategies employed in the study.

Policy	Implementation Level	Implementation time	Approach
The RFS	Federal	2005	SCM’s assumptions
CAFE standards	Federal	2012	Assumptions
Pavley Law	California	2002	SCM’s assumptions
The CAT	Multiple States	2013	SCM (S1 Appendix)

Our identification strategy addresses whether the outcomes of our estimations can be attributed to the effect of the LCFS. We also discuss possible contaminating factors, such as similar state and federal level policies. We discuss strategies to separate and isolate other potential effects from the effect of the LCFS.

There are two regulations which control the carbon dioxide intensity of transportation fuels in California: The Renewable Fuel Standards (RFS) and the LCFS [19]. The RFS was established by the Energy Policy act of 2005, with the purpose of reducing carbon dioxide emissions by increasing the ethanol content of fuels. The RFS is a federal policy, and it does not affect our estimations since the SCM method is immune to pre-intervention changes. The SCM accounts for all trends before the intervention and constructs *counterfactuals* that can adequately reflect all the changes during the pre-intervention period. Therefore, by construction, the SCM helps us to rule out contaminating effects of California’s LCFS arising from the RFS.

Furthermore, California’s clean car law, also known as “Pavley Law”, was adopted in 2002. The Pavley law mostly targets the provision of technical assistance for emission reductions. This law targets cars, and not motor fuels. As discussed before, the SCM incorporates changes before the treatment by constructing counterfactuals, and since the Pavely Law was adopted eight years before the intervention, the SCM provides reliable results [6, 20]. The SCM reduces concerns regarding possible interfering effects from the Pavley Law.

Another potential noise can be the Corporate Average Fuel Economy (CAFE) standards by the federal government, which also target carbon dioxide emissions in the transportation sector. The relationship between the CAFE standards and gas consumption is not precise. It has been argued that the CAFE standards encourage more driving because of a reduction in the per mile cost of driving [21]. The updated CAFE standards have been enforced since 2012, and the standards only apply to 2012 or newer car models. According to the EPA, almost all automakers exceeded the required emission threshold of the CAFE standards. This implies that automakers possibly started to invest in new technologies before 2012. In this case, pre-intervention trends in the CAFE standards are automatically accounted for in the SCM.

Around the time of the LCFS’s adoption, the CAT program had also been heavily discussed in California. The CAT targeted *CO*_2_ emissions for all the industrial sectors of California. To assess whether California’s transportation sector was affected by the CAT, we conduct a separate SCM estimation for *CO*_2_ emissions using all the industrial sectors of California (see S1 Appendix). The results show no measurable effects of the CAT on carbon dioxide emissions.

Another set of potential contaminating factors relates to state-specific consumer preferences in the transportation sector, such as the share of electric and fuel efficient vehicles in the transportation fleet, general economic activity, and political affiliation (We thank anonymous reviewers for pointing our attention to these factors). For instance, one can argue that the increased usage of fuel efficient and electric cars can decrease the total carbon dioxide inventory in California, which eventually may cause an upward bias in our estimations. We address these concerns in S2 Appendix and show that when controlling for these potential factors, our main results still hold. Furthermore, we consider time-variant state-fixed effects (in the SCM), time-invariant fixed effects (in DID), and interactions of linear trends and their squared values with control variables (in Lasso). The effect of the LCFS is robust across all the employed models.

## Model

### Synthetic control method

Policy evaluation has a long history in Economics [22–24]. Early studies mainly employed *ad hoc* techniques to select units of comparison to assess the outcomes of policy changes. For example, the Difference-in-Difference (DID) approach has been extensively used for policy evaluation. Nevertheless, constructing counterfactuals using DID has always been subject to criticism. Since there is not a generally accepted method for choosing non-treated units after a policy intervention, researchers usually rely on *ad hoc* approaches (i.e., selecting surrounding states in our context).

Recent econometric advances provide researchers with robust methods to enhance the construction of counterfactuals for capturing the effect of a policy change. The Synthetic Control (SC) approach is a groundbreaking technique for policy analysis, and it continues to gain the attention of economists [5–7, 25]. The SCM has also been employed in recent environmental economics studies as it offers a framework to control unobservable confounders that vary with time [26]. The novelty of the Synthetic Control Method (SCM) is that as a data-driven approach, it minimizes subjective decisions for constructing counterfactuals. From this perspective, using the SCM to assess the effects of the LCFS enables us to make two major contributions to the energy and environmental policy literature. **First**, most studies have analyzed the LCFS using theoretical models or simulations [27, 28], mostly because analyzing the causal relationship between carbon dioxide emissions and the LCFS poses endogeneity concerns. Studying the consequences of any new policy raises questions about potential omitted variable bias, especially when analyzing the direct outcomes of the policy [20]. The SCM includes time-varying unobserved confounders and thus helps to eliminate the endogeneity arising from omitted variables [6]. The main motivation of using time-variant changes in the SC estimation can be inferred from this question—“What if not only California but also some other states are gradually becoming more environmentally conscious?” If there is a general trend to reduce *CO*_2_ emissions across the United States, then not accounting for time-variant changes may produce an upward bias in the post-treatment estimations. Namely, perhaps the difference between California and other states will turn out to be not significant if we assume that there is a general trend towards more environmentally friendly policies in other states as well. We train our synthetic control model in the period of 1997-2009, and it is reasonable to assume that other U.S. states also made progress in environmental policy-making. Thus, we believe that considering time-variant state-specific changes in the pre-treatment period will also produce robust post-treatment estimates. Therefore, the use of SCM contributes to the related literature by drawing conclusions on the base of the causal relationship between the LCFS and *CO*_2_ emissions. **Second**, most studies analyzing the effect of various policies determine units of comparison for constructing counterfactuals based on previous research and subjective intuition, which may bias the results. The SCM builds counterfactuals without the intervention of the analyst [29].

Let *S* denote the number of all observed units (i.e., states in our context), including California. There are *S* − 1 states as potential donors to construct a counterfactual California. The potential donor pool represents all other states in the United States that can potentially serve as comparison units for constructing a counterfactual California. As mentioned before, some states launched the LCFS initiative around the same time as California, but they never managed to fully implement the initiative. Nevertheless, those states could have been treated with an “anticipation effect”, which in turn may invalidate our synthetic control estimations [6, 20]. To avoid this problem, we eliminate the “falsely treated” states (Washington, Arizona, New Mexico, Minnesota, Illinois, and Oregon) from the potential donor pool. In addition, we drop North-Dakota, due to the oil boom resulting in abnormal population increases [30]. Starting in 2006, Kentucky implemented different programs to incentivize greener transportation fuels. Since this may contaminate the synthetic control estimates, we also drop Kentucky from the potential donor pool. Thus, the actual donor pool includes only those states that have not been affected by the LCFS or similar policies during the pre-intervention period. Therefore, to determine the actual donor pool, we exclude states with identical or similar regulations during the same time frame. After eliminating “ineligible” states, we end up with 42 states in the potential donor pool.

Denote the final time period in our data as *T*, where *T*_0_ is the last period before the policy is implemented. Then the post-intervention period covers [*T*_0_ + 1, *T*]. Let $Y_{st}^{NR}$ be the emission level for state *s* with No-Regulation (NR) in period *t* and $Y_{st}^{R}$ be the emission level for state *s* in period *t* with Regulation (R). Here *t* falls in [1, *T*]. Then we have two possible scenarios:

$$\begin{array}{c} Y_{st}=\{\begin{array}{cc} Y_{st}^{NR} & in\;the\;absence\;of\;LCFS\\ Y_{st}^{R}\equiv Y_{st}^{NR}+\tau_{st}D_{st} & in\;the\;presence\;of\;LCFS \end{array} \end{array}$$ (2)

$\tau_{st}=(Y_{st}^{R}-Y_{st}^{NR})$ is the effect of the Law for state *s* at time *t*, where *D*_*st*_ = 1 (i.e., the treatment) if *t* > *T*_0_ and *D*_*st*_ = 0 if *t* ≤ *T*_0_. For the treated state (i.e., for California) we observe $Y_{st}^{R}$, but not $Y_{st}^{NR}$ in the post-intervention period. Therefore, our challenge is to construct an appropriate counterfactual for California for the period (*T*_0_, *T*]. A linear factor model is used to construct the California counterfactual:

$$\begin{array}{c} Y_{st}^{NR}\equiv \alpha_{t}+\theta_{t}Z_{s}+\lambda_{t}\mu_{s}+\epsilon_{st} \end{array}$$ (3)

where *α*_*t*_ is an unknown common factor with constant factors across states, *Z*_*s*_ (*r* × 1) is a vector of observed variables for each state (we select variables which are not affected by the regulation), *θ*_*t*_ is a (1 × *r*) vector of parameters that have to be estimated, *μ*_*s*_ is a (*F* × 1) vector of unknown parameters, λ_*t*_ is a (1 × *F*) vector of unobserved common factors, and *ϵ*_*st*_ are state specific shocks, and assumed to have a zero mean. As [6] note, this specification allows the effects of confounding unobserved characteristics to be time variant. Moreover, the traditional DID approach only allows time-invariant unobserved characteristics (λ*μ*_*s*_). We discuss the DID model in the next section.

California can be expressed as a weighted average of other states in the donor pool. Therefore, synthetic California can be constructed using a (*J* × 1) vector of weights: *W* = (*w*_1_, …, *w*_*J*_)′. In *W*, we follow the usual assumptions for weight: *w*_*j*_ ≥ 0 for *j* = 1, …, *J* and $\sum_{j=1}^{J}w_{j}=1$ [6, 25]. Thus, each potential synthetic control state is:

$$\begin{array}{c} \sum_{j=1}^{J}w_{j}Y_{jt}=\alpha_{t}+\theta_{t}\sum_{j=1}^{J}w_{j}Z_{j}+\lambda_{t}\sum_{j=1}^{J}w_{j}\mu_{j}+\sum_{j=1}^{J}w_{j}\epsilon_{jt} \end{array}$$ (4)

For the optimal weights ${\left(w_{1}^{*},...,w_{J}^{*}\right)}^{\prime }$ we can get [6]:

$$\begin{array}{c} \sum_{j=1}^{J}w_{j}^{*}Y_{j1}=Y_{11,\;\;}\sum_{j=1}^{J}w_{j}^{*}Y_{j2}=Y_{12,\;\;...,} \end{array}$$ (5)

$$\begin{array}{c} \sum_{j=1}^{J}w_{j}^{*}Y_{jT_{0}}=Y_{1T_{0}}\;and\;\sum_{j=1}^{J}w_{j}^{*}Z_{j}=Z_{1} \end{array}$$ (6)

Here the major hurdle is to determine a set of weights for the emission level of the donor pool that closely mimics the emission level of California. If this is the case, then there should also be a small bias in the post-intervention period. Then:

$$\begin{array}{c} {\hat{\tau }}_{California,t}=Y_{California,t}-\sum_{j=1}^{J}w_{j}^{*}Y_{jt} \end{array}$$ (7)

where *t* ∈ {*T*_0_ + 1, …, *T*}.

$\text{K}={\left(k_{1},..,k_{T_{0}}\right)}^{\prime }$ is a linear combination of pre-intervention emission levels: ${\bar{Y}}_{j}^{K}=\sum_{m=1}^{T_{0}}k_{m}Y_{jm}$ where *j* ∈ {1, .., *J*} and *j* = *J* + 1 is the treated state (California). Let *X*_1_ be ${\left(Z_{1}^{{}^{\prime }},{\bar{Y}}_{1}^{K_{1}},\ldots ,{\bar{Y}}_{1}^{K_{M}}\right)}^{\prime }$, a vector of pre-treatment variables that closely represent California and *X*_0_ includes the same set of pre-treatment indicators for each donor state. Then:

$$min_{W^{*}\in W}\sqrt{{\left(X_{1}-X_{0}W\right)}^{{}^{\prime }}V\left(X_{1}-X_{0}W\right)}$$ (8)

where *V* is a symmetric, diagonal and positive-definite matrix that minimizes the mean square prediction error (MSPE) of the emission level in the pre-intervention period.

### Differences-in-Differences

**Fig 1:**
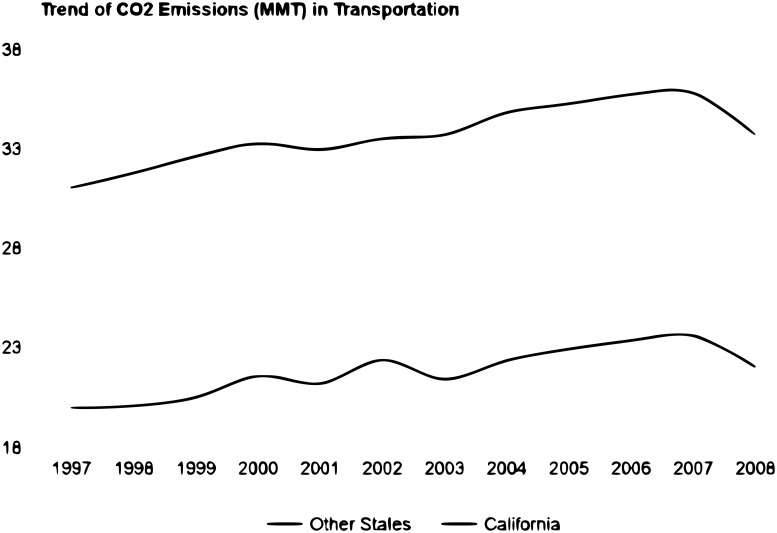
Testing the validity of the parallel trend assumption for the DID estimation.

DID is used as a robustness measure to validate the SCM results. Fig 1 depicts the average *CO*_2_ emissions in the transportation sector (in Million Metric tons) for all states (except California) with the blue line and California with the red line. Fig 1 helps to check the “parallel trends” assumption that should be validated in the preintervention period. Furthermore, we scaled down California’s observations ten times for comparison purposes. Of course, this does not change the trend. The Y and X axis represent emissions and years, respectively. The graph shows that both California and “All Other States” have almost identical trends during the pre-intervention period. In the DID literature, this is known as the “parallel trends” assumption, and it is the key for identification. Since in our case, the parallel trends assumption is satisfied, then DID can be used as a robustness check for our results [31].

A simple OLS representation of DID is used:

$$\begin{array}{c} Y_{st}=\alpha d_{st}+X_{st}^{{}^{\prime }}\beta +\theta_{s}+\gamma_{t}+\eta_{st} \end{array}$$ (9)

where *d*_*st*_ is a policy indicator and we are interested in estimating *α*. *X*_*st*_ is a vector of control variables. In line with [32], *η*_*st*_ is state-time random effects. *θ*_*s*_ is a time-invariant fixed effect for state *s* and *γ*_*t*_ is a time fixed effect that is common across all states but varies across time *t* = 1, …, *T* [33]. In our setting, only California experiences the policy change and the policy change is permanent. All other donor states have time-invariant policies: *d*_*j*1_ = … = *d*_*jT*_ = 0 for *j* = 1, …, *J*. Within this framework, as discussed above the OLS DID estimation yields consistent results [31].

### Least absolute shrinkage and selection operator (Lasso)

The promise of this paper is to shed light on effective approaches in energy regulations. We study the case of California employing two different econometric methods. Thus, our analysis provides a strong foundation for internal validity. But to what extent do our results have external validity? It has been argued that California is an outlier when it comes to environmental and energy policies; in order to obtain nationwide policy insights the experience of other states also needs to be studied [34]. Furthermore, in the treatment effects literature, it is conventionally assumed that treatment is randomly assigned [35, 36]. There is no random assignment in our study. Hence, it can be easily argued that the treatment in our study is endogenous and it is related to other variables (which are mostly unobserved) and with environmental and energy policy trends in California. Controlling for other variables can mitigate the problem [35, 36]. However, this approach opens the door to another concern: selecting control variables. Researchers usually adopt *ad hoc* intuition (mostly guided by economic theory) in selecting control variables. Although we can also add control variables, our *ad hoc* control variable selection might be subject to criticism as well. Therefore, we turn to machine learning and its increasing application in economic studies. [8] propose the Lasso method for selecting not only the control variables but also their lags, differences, and interaction terms.

We also test our results with the Lasso “post-double-selection” method. The main purpose of using Lasso is to validate the choice of control variables and to account for potential endogeneity in the policy treatment. We are specifically concerned with the lags of our control variables and their interaction with trends. [8] construct 284 controls, and the post-double-selection method selects only eight for their abortion equation and none for the crime equation. We follow [8] in setting our Lasso “post-double-selection” method. We use a partial linear model:

$$\begin{array}{c} y_{i}=d_{i}\alpha_{0}+g(z_{i})+\zeta_{i},\;\;\;\;\;\;E[\zeta_{i}|z_{i},d_{i}]=0 \end{array}$$ (10)

$$\begin{array}{c} d_{i}=m(z_{i})+v_{i},\;\;\;\;\;\;\;\;\;\;E[v_{i}|z_{i}]=0 \end{array}$$ (11)

where *y*_*i*_ is the dependent variable and *d*_*i*_ is the policy variable. We are interested in estimating *α*_0_ correctly while controlling for other *z*_*i*_ covariates. *ζ*_*i*_ and *v*_*i*_ are disturbances. The main novelty of this model is to conduct inference for the treatment effect *α*_0_. This procedure selects a set of variables from *p* potential regressors *x*_*i*_ = *P*(*z*_*i*_), which consists of *z*_*i*_ and their transformations, to approximate *g*(*z*_*i*_). The method assumes that once ex-ante unknown variables from *x*_*i*_ are controlled, we can treat *d*_*i*_ as exogenous. It implies that linear combinations of unknown variables help to approximate *g*(*z*_*i*_) and *E*[*d*_*i*_|*z*_*i*_] = *m*(*z*_*i*_). Thus our problem boils down to finding an appropriate set of variables for estimating *α*_0_. The Lasso post-double-selection is implemented using the following steps:

1. During the first step, the control variables $Z_{1}$ that may help to predict treatment *d*_*i*_ are selected. This step ensures the validity of post-model-selection-inference by accounting for potential confounding factors.
2. In the second step, the primary goal is to select additional variables $Z_{2}$ that predict *y*_*i*_. This step also tries to detect confounding factors that may have been omitted in the first step.
3. In the third step, we regress *y*_*i*_ on *d*_*i*_ and Z. Here $Z=Z_{1}\cup Z_{2}$.

We follow [8] and use first-difference of the dependent variable using an OLS regression. Please note that control variables include first-difference variables as well.

## Data

We use public data sources (see Table 2) in our analysis and due to data availability issues our focus period is 1997-2014. Our primary interest in this study is carbon dioxide emissions in the transportation sector in California. We obtained annual state-level carbon dioxide (CO2) emission inventories data from the U.S. Environmental Protection Agency. The dataset provides emissions information from fossil fuel combustion and breaks down emission inventories by end-use sector (commercial, industrial, residential, transportation, and electric power). The data are in million metric tons of carbon dioxide (MMTCO2). The annual number of total registered vehicles, length of all public roads (in miles), number of miles driven and gas tax rates (cents per gallon) for each state were obtained from the U.S. Department of Transportation Federal Highway Administration. Annual motor fuel consumption (in barrels) and motor fuel expenditures (USD) for each state were obtained from the State Energy Data System of the U.S. Energy Information Administration. The partisan composition statistics of state legislatures was collected from National Conference of State Legislatures. Finally, annual state-level GDP (USD) and population data were obtained from the Regional Economic Accounts of the Bureau of Economic Analysis.

**Table 2:** Summary statistics of the data.

Variables	N	Mean	Std. Dev.	Min	Max
Emissions per mile in the transportation sector (MMTCO2e/mile)	774	7.95e-10	4.61e-10	3.13e-10	4.36e-09
per capita road length (miles)	774	0.02	0.02	0.00	0.11
per capita GDP (USD)	774	44307.55	19249.84	21302.74	176618.10
per capita residential emissions (MMTCO2e)	774	1.37e-06	6.82e-07	4.20e-08	3.95e-06
per capita number of vehicles	774	0.81	0.18	.09	1.37
gas taxes (cents/gallon)	774	20.99	5.78	7.50	39.50
Total emissions in the transportation sector (MMTCO2e)	774	37.81	43.21	1.04	236.16
Total emissions in residential emission (MMTCO2e)	774	6.94	7.91	0.05	39.40
Total emissions in electricity production (MMTCO2e)	774	42.89	43.05	0	237.77
Total road length (miles)	774	76791.65	56544.44	1421	313596
gas consumption (USD)	774	6.21e+07	6.92e+07	2238000	3.77e+08
Total driven miles (miles)	774	5.82e+10	6.26e+10	3.31e+09	3.33e+11
GDP (USD)	774	2.60e+11	3.38e+11	1.48e+10	2.32e+12

Our outcomes of interest are *CO*_2_ emissions per mile in the transportation sector (MMTCO2e/mile) (in the SCM) and total emissions in the transportation sector (MMTCO2e) (in DID, and Lasso). We control *per capita* road length (miles), *per capita* GDP (USD), *per capita* residential emissions (MMTCO2e), *per capita* number of vehicles, gas taxes (cents/gallon) in the synthetic control analysis. Furthermore, we control total road length (miles), the size of population, total driven miles (miles), the number of vehicles, total emissions from residential areas, state GDP (MMTCO2e), gas taxes (cents/gallon) in DID and Lasso estimations.

Transportation emission data covers on-road vehicles, which is useful to measure the target of the LCFS. In our SCM estimations, we use “emissions per mile” as the outcome variable, since most gasoline consumption and pollution policy studies focus on emissions per mile estimates (EPM) [21, 37]. However, the amount of miles driven is a complement of leisure, and it is strongly correlated with economic growth [21]. To eliminate other potential effects in the denominator of the EPM measure, we use 1997 as the baseline year. All subsequent emission data points are normalized by the number of miles driven in 1997. We also conducted sensitivity analyses to assess how changing the baseline year affects our estimations. After 2004, several economic and policy events can influence the number of driven miles (adoption of RFS in 2005, the financial crisis in 2007, etc.). Hence, we changed the baseline year to 2004 and replicated our calculations. Furthermore, we also used average miles driven for each state in the 1997-2004 period as the denominator for EPM. In all the sensitivity analyses the effects were almost identical. We conclude that selecting 1997 as the baseline did not affect the results.

The second reason for normalizing variables in the SCM is to account for the size of California. When we apply the SCM, none of the states from our donor pool can closely mimic California. Because California is not comparable to any other states regarding emissions and other variables. The SCM with level variables assigns a 100% weight to Texas (i.e., Texas is chosen as synthetic California) and even Texas does not closely represent California. [6] discuss a similar issue in their placebo test for New Hampshire cigarette sales application. New Hampshire has the highest *per capita* cigarette sales in their sample, and they cannot find any single state or a combination of states that can reproduce New Hampshire’s time series of *per capita* cigarette sales during the pre-intervention period. [6] used *per capita* cigarette consumption for California as the dependent variable in their SCM estimations. We follow a similar approach in our SCM estimations. However, we use overall emissions in the DID and Lasso estimations. Our controls in DID and Lasso estimations are state public road length, population, amount of annual miles driven, total number of vehicles, residential emissions (MMT), GDP and gas tax values.

We pay particular attention to the selection of the control variables. Since our main model is the SCM, we comply with its requirements. We used the same set of control variables in DID and Lasso estimations to maintain the comparability between our robustness checking analyses and our primary model. The major requirement of the SCM is selecting only those control variables which have not been affected by the treatment [6]. Furthermore, we also consider that transportation emissions are the result of consumption decisions of households, which are strongly affected by prices and income [38]. This is the rationale for using *per capita* GDP and state tax rates. Furthermore, emissions can be driven by other factors, such as the number of vehicles in the state, and the length of public roads. For example, the construction of new public roads creates new residential areas (or vice versa), and this can increase the number of annual miles driven and subsequently emissions. Therefore, we use the annual *per capita* number of registered vehicles and *per capita* miles driven as controls. We also use *per capita* residential carbon dioxide emissions as a control variable. This variable captures consumer sensitivity to emissions, which is a crucial factor in energy consumption [39].

## Estimation results

### Outcomes of the SCM estimation

**Fig 2:**
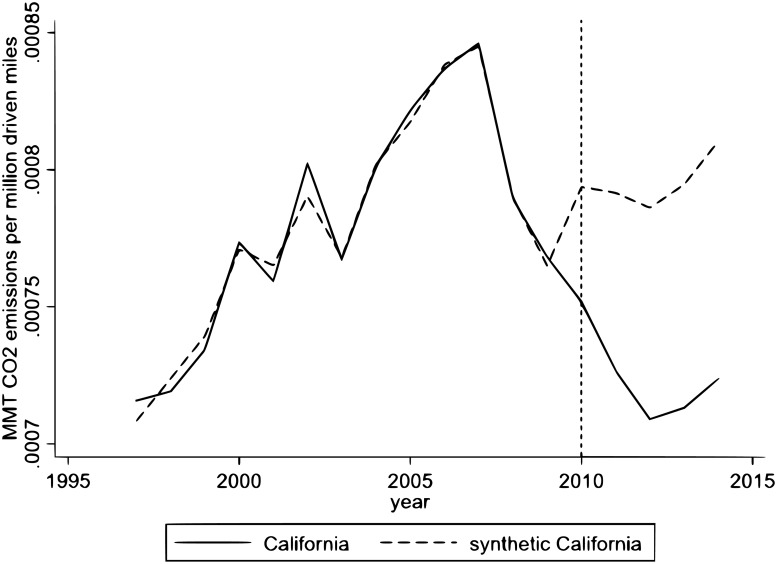
Results of the synthetic control estimation to measure the effect of the LCFS on emissions.

We construct “synthetic California” as a convex combination of the donor states [6]. The weights of donor states are chosen to minimize the distance between California and synthetic California in the pre-intervention period. Fig 2 shows the per mile driven *CO*_2_ emissions from the transportation sector in California and synthetic California during the pre-intervention and the post-intervention periods.

It is evident that during the pre-intervention period, there is almost a perfect overlap between synthetic California and actual California. The deviation between synthetic California and California started a year before the LCFS was implemented. This result indicates signs of an “anticipation effect”, with the deviation between synthetic and actual California becoming larger as time progresses. In fact, the average Californian biodiesel production capacity increased from 4.96 million gallons in 2008 to 9.46 million gallons in 2009 (see http://www.energy.ca.gov/2009-ALT-1/documents/2009-09-14_workshop/2009-09-14+15_presentations/CEC_McCormack-McKinney_%20Status_of_In-state_Production.pdf). This result confirms that California producers started adjusting their production according to the LCFS in 2009 and explains the observed “anticipation effect”.

Table 3 compares the pre-intervention characteristics of California with synthetic California. It should be noted that in synthetic California, lags of the dependent variables *(per capita* residential emissions, *per capita* vehicles and gas taxes) behave very well in terms of closely mimicking their values for California. However, *per capita* GDP and *per capita* road length do not have good predicted values. [25] show that, in spite of this mismatch, if synthetic and real outcome variables (i.e., the per mile driven *CO*_2_ emissions in our case) are very close in the pre-intervention period (which is what we observe in Fig 2), the results of the SCM are valid. Fig 2 shows that synthetic California very closely reproduces California in the pre-intervention period. Therefore, the mismatch among some control variables and their predictors is not a cause for concern. Moreover, in S3 Appendix, we drop *per capita* GDP and *per capita* road length and re-estimate the SCM. The results show that dropping those variables does not change the results of our estimations. Nevertheless, we keep them as a part of our model to make it comparable to the DID and Lasso estimations.

**Table 3:** Emissions per mile (EPM) predictor means before the LCFS.

Variables	Actual California	Synthetic California
EPM (1997) (MMTCO2e/mile)	7.16E-10	7.08e-10
EPM (1998) (MMTCO2e/mile)	7.19E-10	7.24e-10
EPM (2000) (MMTCO2e/mile)	7.74E-10	7.71e-10
EPM (1999(1)2001) (MMTCO2e/mile)	7.56E-10	7.58E-10
EPM (2001&2002) (MMTCO2e/mile)	7.81E-10	7.78E-10
EPM(2003) ((MMTCO2e/mile)	7.67E-10	7.68E-10
EPM (2004) (MMTCO2e/mile)	8.01E-10	8.02E-10
EPM (2006) (MMTCO2e/mile)	8.37E-10	8.38E-10
EPM (2007) (MMTCO2e/mile)	8.46E-10	8.45E-10
EPM (2008) (MMTCO2e/mile)	7.89E-10	7.90E-10
EPM (2009) (MMTCO2e/mile)	7.68E-10	7.65E-10
Per capita road length (miles)	0.01	0.04
Per capita GDP (in USD)	44647.85	34518.43
Per capita residential emissions (2009) (MMTCO2e)	7.65e-07	7.51e-07
Per capita residential emissions (2003) (MMTCO2e)	8.03e-07	8.34e-07
Per capita number of vehicles (2000)	0.81	0.86
Per capita number of vehicles (2009)	0.93	0.92
Fuel taxes (cents/gallon)	18.00	18.88

We also provide the list of donor states with non-zero weights by the SCM estimations. Table 4 depicts donor states and their weights in synthetic California. Table 4 shows that around 27% of synthetic California has been constructed from Florida. Alabama (∼19%), South Dakota (∼18%), Texas (∼12%), Oklahoma (∼9.5%) and Montana (∼9%) are major contributors to synthetic California; these states together constitute nearly 84% of synthetic California. Hawaii, Rhode Island, and Maine have weights that are less than 1% in synthetic California (Below we show that the exclusion of Hawaii, Rhode Island, and Maine does not affect the results).

**Table 4:** Weights from the main synthetic control estimation to construct synthetic California.

Donor States	Weights	Donor States	Weights
Florida	0.267	Montana	0.087
Hawaii	0.001	Alabama	0.19
Louisiana	0.028	Utah	0.022
Maine	0.002	Oklahoma	0.095
Rhode Island	0.004	South Dakota	0.183
Texas	0.121		

Note: Other states were assigned zero weights and therefore were omitted from Table 4.

As a robustness check, we ran an additional SC estimation with non-eligible (or falsely treated) states (detailed results are available upon request). Even with the falsely treated states included in the donor pool, we replicate the results of the major SC estimation. This result suggests that those states did not experience significant changes in the studied period. We want to make it clear that we do not conclude anything about *CO*_2_ emissions in the transportation sectors of falsely treated states and this discussion should be a separate research study. However, we follow the Synthetic Control literature and drop falsely treated states from our major estimations as a precautionary measure.

This data-driven method provides a more solid ground in the estimations while avoiding “extreme counterfactuals”, i.e., the counterfactuals which are outside the convex hull of the data [6, 40]. Conventional regression methods assign weights to all units in the control group (donor pool in our case). As a result, some units are assigned positive weights, while other units can have negative weights. It is possible for positive and negative weights to counterbalance one another [25]. Thus, conventional methods may produce a net effect that might not represent the real effect of the LCFS.

It is also noteworthy that all other states, including California’s neighboring states, have been assigned zero weights. Some studies discuss a “linkage effect” or a “reshuffle effect” of energy and environmental policies [41, 42]. Regulatory goals for environmental and energy markets can be undermined by reshuffling “dirty” productions to adjacent jurisdictions. Since there are no positive weights for California’s neighboring states, in our case, the synthetic control analysis is less likely to be contaminated by these effects. Furthermore, this outcome provides evidence for the validity of our identification strategy.

We conduct several placebo tests to validate the results. We re-estimate the SCM several times while randomly choosing one of the states from the donor pool to act as the treated state. The logic behind this test is to construct the inference for the primary SCM analysis by observing whether other states had similar effects. If other states experienced effects similar in magnitude to California, then we can rule out the impact of the LCFS on the transportation emissions in California.

We proceeded in three steps. In the first step, we excluded those states from our placebo sample that had pre-intervention MSPE more than ten times the MSPE of California. We also used a five-time cutoff and a two-time cutoff. After applying the final exclusion criteria, there were 23 states in the sample.

**Fig 3:**
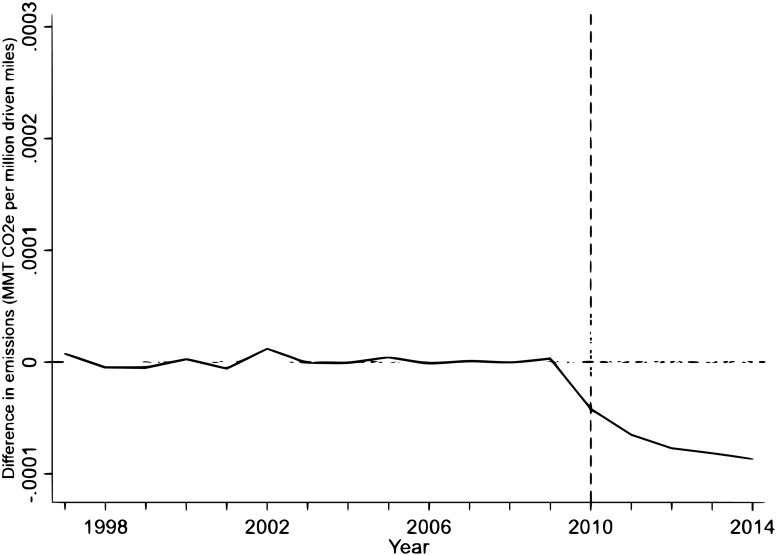
Placebo test of the synthetic control estimation to measure the effect of the LCFS on emissions.

Fig 3 plots the placebo test (for a two-times cutoff). California (red line) stands out from the other 23 states regarding a reduction in carbon dioxide emissions in the transportation sector. So, we conclude that the impact of the LCFS is robust.

The SCM works well if the pre-intervention period Root Mean Squared Prediction Error (RMSPE) is low. RMSPE for the pre-intervention period is calculated as:

$$\begin{array}{c} \Omega ={\left(\frac{1}{T_{0}}\sum_{t=1}^{T_{0}}(Y_{1t}-\sum_{j=1}^{J+1}w_{j}Y_{jt})\right)}^{1/2} \end{array}$$ (12)

**Fig 4:**
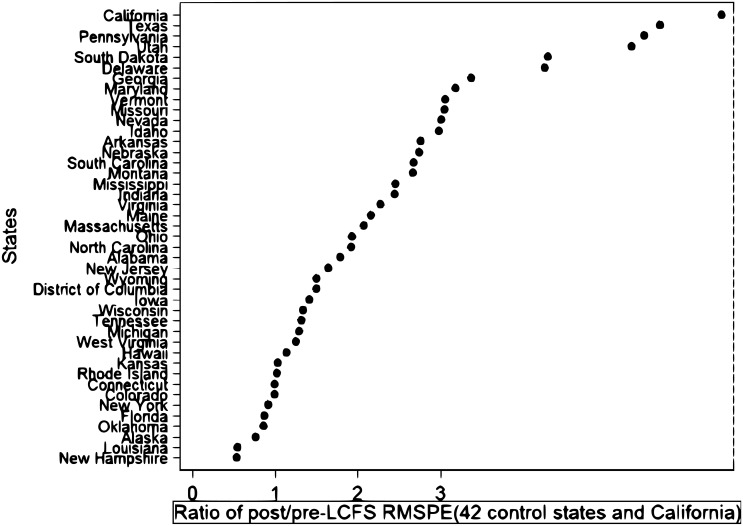
The post-pre RMSPE test of the synthetic control estimation to measure the effect of the LCFS on emissions.

The ratio of the post-intervention RMSPE to the pre-intervention RMSPE compares the treated state and its synthetic counterfactual in the post-intervention period relative to the pre-intervention period. In the presence of a treatment effect, it is expected that the treated state will have the highest ratio of the post-RMSPE to the pre-RMSPE. For this reason, in Fig 4 we plot the ratios of post/pre RMPSE for California and other states, following [6].

Fig 4 shows that the value of California’s post/pre RMSPE stands out from all the other donor states. The ratio of post/pre RMSPE is mainly used for inference purposes to calculate the *p-value* for the SCM estimation. The *p-value* can be constructed as:

$$\begin{array}{c} p-value=\frac{\sum_{j=1}^{J+1}1(\Omega_{j}\geq \Omega_{California})}{J+1} \end{array}$$ (13)

where Ω_*California*_ is the ratio of post/pre RMSPE for California. For each *j* = 1, …, *J* state and California, we calculate *Ω* values and compare them to values obtained for California. The indicator function in the numerator counts the number of states that have *Ω* values at least as high as those of California. Then, this number is divided by the total number of states in the sample. It is evident from Fig 4 that only California itself can pass this threshold; thus our *p-value* for the SCM estimation is *p* − *value* = 1/43 = 0.023. Therefore, the estimated effect of the LCFS for California is statistically significant.

We also calculate the average treatment effect for California by averaging the treatment effects for all the post-intervention periods (-19.7 MMT *CO*_2_ or a 9.85% reduction). This estimate is very similar to the estimates obtained from the DID and Lasso estimations (we discuss the results of those estimations in the next sections).

**Fig 5:**
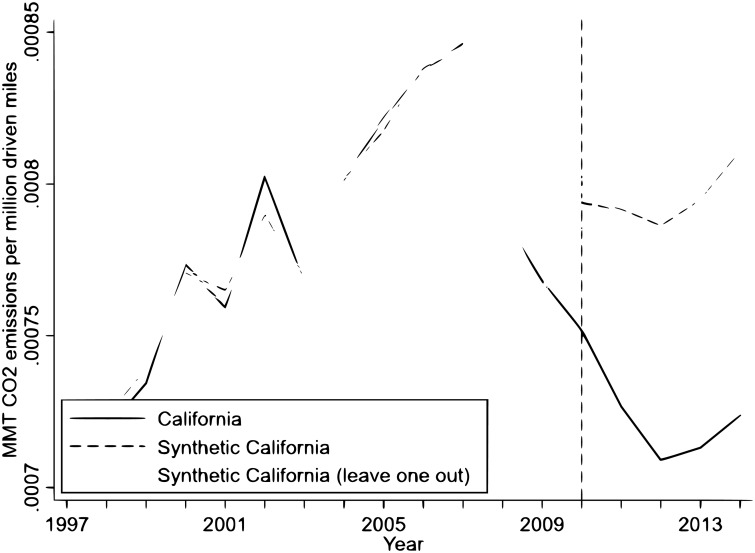
Leave-one-out test of the synthetic control estimation to measure the effect of the LCFS on emissions.

We also conduct the leave-one-out test and show the results in Fig 5 [25]. The purpose of this test is to assess to what extent the synthetic construction for California is sensitive to a particular donor state that received a non-zero weight. Ideally, the synthetic construction and eventually the treatment effect should not be sensitive to the exclusion of any donor state. Fig 5 shows the result of the leave-one-out test. In all cases, we observe a significant treatment effect. Thus our result is not sensitive to the exclusion of any donor state.

### Outcomes of DID

Table 5 shows the primary results from the Difference-in-Difference estimations. Since we dropped states which intended to adopt similar laws, we assume that observations for each remaining state are independent. We expect that observations are clustered (for each state), i.e., correlated in the same cluster and independent across clusters [43].

**Table 5:** Difference-in-Differences estimations to measure the effect of the LCFS.

	OLS (Robust S.E.) Emissions	OLS (Robust S.E.) Emissions	OLS (Clustered S.E.) Emissions	OLS (Clustered S.E.) Emissions
Treatment	-14.27** (3.31)	-21.19*** (3.59)	-14.27*** (0.47)	-21.19*** (2.36)
Year	Yes	Yes	Yes	Yes
State	Yes	Yes	Yes	Yes
Controls	No	Yes	No	Yes
Constant	30.31*** (0.70)	19.33*** (4.17)	30.31*** (0.73)	19.33*** (4.68)
N	774	774	774	774

Note: Standard errors in parenthesis. In all tables, *, ** and *** indicate 5%, 1% and 0.1% significance respectively

Our dependent variable is *CO*_2_ emissions in the transportation sector (MMT) and we control for public road length, population, number of annual miles driven, number of registered vehicles, *CO*_2_ emissions in the residential sector, state-level GDP and state level fuel taxes. The model with controls predicts that the adoption of the LCFS in the transportation sector decreased emissions about 21.19 Million Metric Tons. The magnitude of the reduction represents around 10% of total transportation emissions.

**Fig 6:**
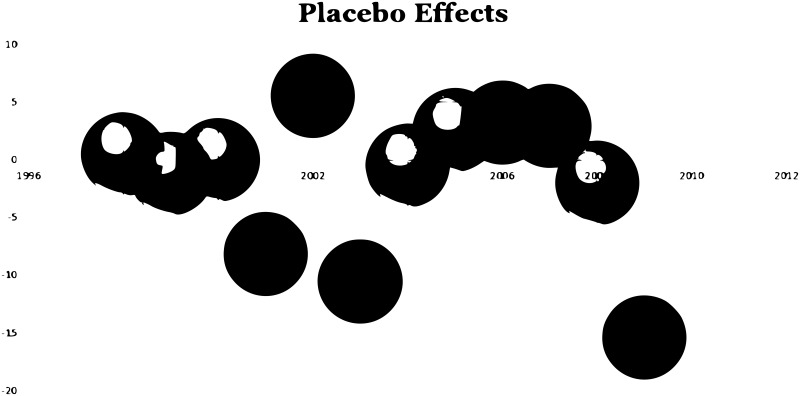
Placebo test for the DID estimation to measure the effect of the LCFS on emissions.

We also conducted a placebo test for the DID estimations. Our primary concern is whether a placebo effect was observed before 2010 and in line with [31], we remove the 2011-2014 treatment period from our sample to test for it. We conducted a series of placebo regressions starting in 1998. For instance, in 1998, we specified 1998 as the treatment year and 1997 as the control year. Fig 6 shows the placebo effects in California for each placebo test. The Red bubbles represent statistically significant placebo effects. We observe significant and large placebo effects around 2002. This result is mainly due to the implementation of California “Pavley Law” which controlled carbon dioxide emissions from transportation vehicles by setting technical emission control measures. Since the “Pavley Law” was successful in California, it was later adopted by other states.

Except for 2009, the placebo effects in other years are not significant or only weakly significant with magnitudes near zero. According to Fig 6, the placebo treatment effect in 2009 stands out from the other results. This result is in line with the results of the SCM analysis which also revealed an “anticipation effect” in 2009. Nevertheless, the placebo effect in 2009 is around (-15 MMT), which is lower than the treatment effect (-21.19 MMT or 10.95% reduction in 2010). Thus the placebo test validates the DID results.

### Outcomes of Lasso

Following [8] the Lasso model addressed concerns about trends and their interactions with lags and first differences. Thus, for each control variable we generated first difference, first difference squared, one period lag, squared of lag and their interactions with the trend and squared trend. Furthermore, we also included time dummies for each year. The Lasso estimation selected 84 control variables with 12 time dummies as controls; hence they were all included as Lasso controls into the regression. We used the clustered errors technique in the Lasso estimations (The Lasso controls are available upon request).

Table 6 shows the results of the Lasso estimations. The Lasso method detects a 26.56 MMT (or 13.28%) reduction in carbon dioxide emissions in the transportation sector of California. Hence, the machine learning technique yields a similar result, while including all possible control variable combinations. This result is similar in magnitude to the previous results using the DID and SCM. In conclusion, the Lasso estimation strengthens the result that in the post-intervention period the LCFS significantly decreased *CO*_2_ emissions in the transportation sector of California.

**Table 6:** Lasso post double selection to measure the effect of the LCFS.

	Emissions (first difference)	Emissions (first difference)	Emissions (first difference)
Treatment	-3.786*** (0.18)	-3.020** (0.90)	-26.56** (9.38)
Year	Yes	Yes	Yes
State	Yes	Yes	Yes
Controls	No	Yes	Yes
Lasso Controls	No	No	Yes
Constant	0.053 (0.05)	-0.188 (0.33)	-1.009* (0.38)
N	688	688	688

Note: Standard errors in parenthesis. In all tables, *, ** and *** indicate 5%, 1% and 0.1% significance respectively

Overall, all three methods show around a 10% reduction in carbon dioxide emissions. We used Carbon Dioxide Information Analysis Center’s conversion tables (see http://cdiac.ornl.gov/pns/convert.html) and according to those tables, 21.19 MMT decrease in *CO*_2_ emissions (the outcome of the DID estimations) is equal to a 0.002 ppb *CO*_2_ reduction. According to [44], the amount of *CO*_2_ reduction in the atmosphere can increase worker’s productivity by 0.011% in California. Assuming a linear relationship between worker’s productivity and GDP, then this translates into an annual 270 Million USD increase in the GDP of California (using California’s 2015 GDP).

## Conclusion

Increased environmental concerns have resulted in the application of new economic and energy policy tools. “Bottom-up” sector-specific *CO*_2_ mitigation policies, such as setting emission standards covering all stages of production have gained the attention of policymakers. California’s Low Carbon Fuel Standards (LCFS) to motor fuels is the very first attempt to apply this new policy approach at the state level. The LCFS is an entirely new approach in environmental policy-making that aims at limiting the carbon dioxide footprint of on-road vehicles with the help of life-cycle accounting principles. California was the first state to implement the LCFS and there is a vast literature on procedural details of this policy [3, 4, 16, 19, 28, 45, 46]. However, as [3] point out, it is very important to quantify the actual *CO*_2_ reductions under low carbon standards. This study is one of the first attempts to rigorously measure the effect of the LCFS on carbon dioxide emissions. We conduct our analysis by applying different robustness checking tools and empirical models which have been advised by previous studies [3]. We analyzed the effects of the LCFS using three different estimation techniques. The Synthetic Control Method (SCM) constitutes the center of our estimations because it minimizes subjective decisions by using a data-driven approach to construct counterfactuals. Furthermore, the SCM includes time-varying unobserved confounders and thus helps to eliminate endogeneity from potentially omitted variables; thus have been implemented in recent environmental economics studies [26]. We also used the Difference-in-Differences and Lasso machine learning techniques as robustness checks for our results. The three different methods yield similar results confirming that the Low Carbon Fuel Standards have reduced carbon dioxide emissions in California’s Transportation sector by about 10% (see Table 7).

**Table 7:** Recap of estimations to measure the effet of the LCFS.

	Synthetic Control	Difference in Differences	Lasso
Effect of the LCFS to CO2 emissions in Transportation (MMT)	-19.7** (or 9.85% reduction)	-21.19*** (or 10.95% reduction)	-26.56*** (or 13.28% reduction)

Note: Standard errors in parenthesis. In all tables, *, ** and *** indicate 5%, 1% and 0.1% significance respectively

However, this reduction is attributable to the first phase of the policy, and it is very crucial to conduct similar analyses for the following stages, since they have higher compliance levels. Moreover, future researchers should employ a more holistic approach and analyze the potential interaction of the LCFS with other related programs [47, 48]. Studying these interactions will contribute to improve our understanding of the overall effect of low carbon fuel standards in curbing carbon dioxide emissions [3, 11]. Future research may take advantage of micro-level data to assess the effects of the LCFS on health indicators of California residents. Furthermore, other modeling approaches to analyze the LCFS can also study the relationship between the LCFS and green technology advances [48, 49].

## Supporting information

S1 Appendix (PDF)

S2 Appendix (PDF)

S3 Appendix (PDF)

S1 Dataset (DTA)
