# Correction: Does California’s Low Carbon Fuel Standards reduce carbon dioxide emissions?

**DOI:** 10.1371/journal.pone.0210906

**Published:** 2019-01-10

**Authors:** Samir Huseynov, Marco A. Palma

Figs 1–6 are incorrect. The authors have provided corrected versions here.

**Fig 1:**
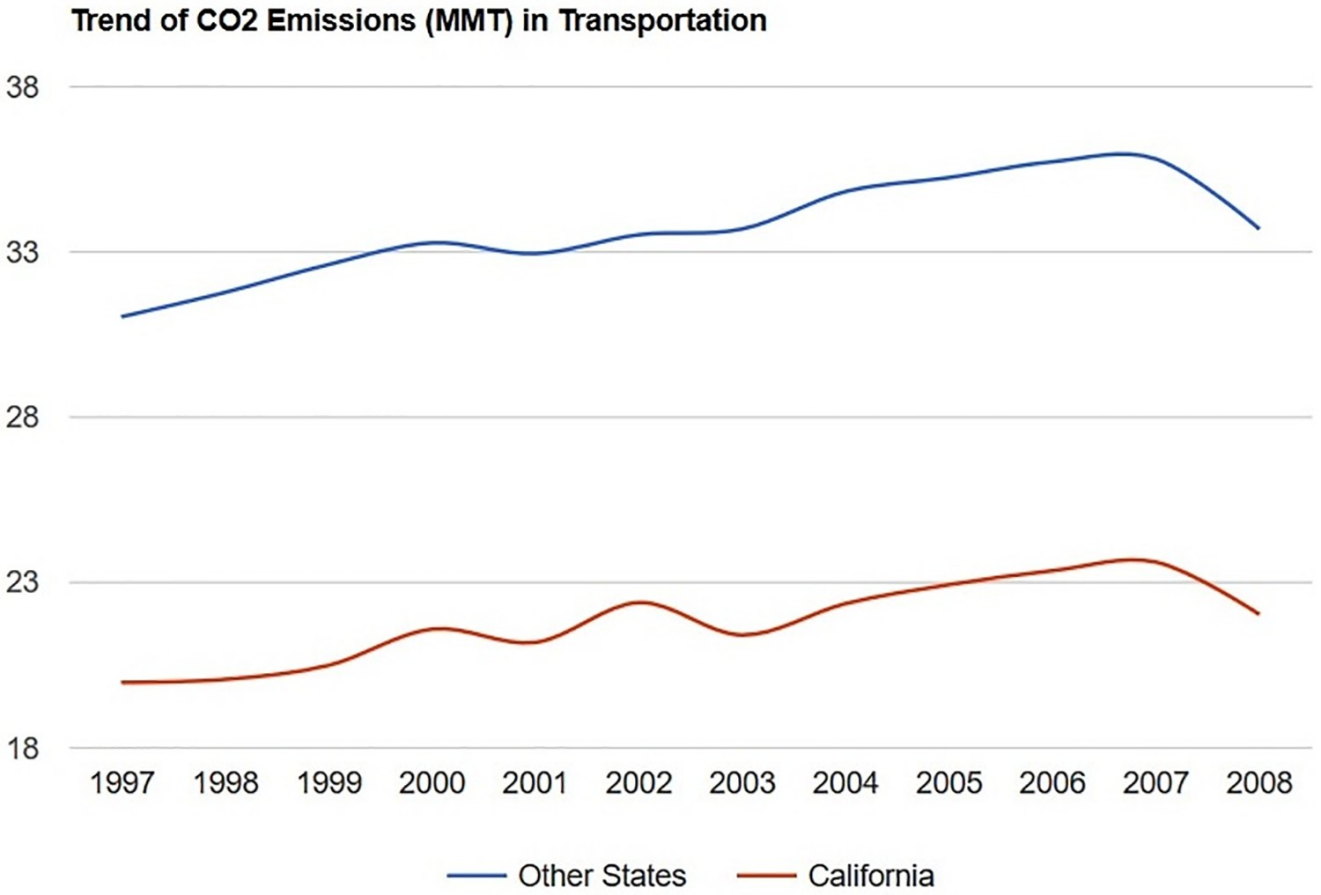
Testing the validity of the parallel trend assumption for the DID estimation.

**Fig 2:**
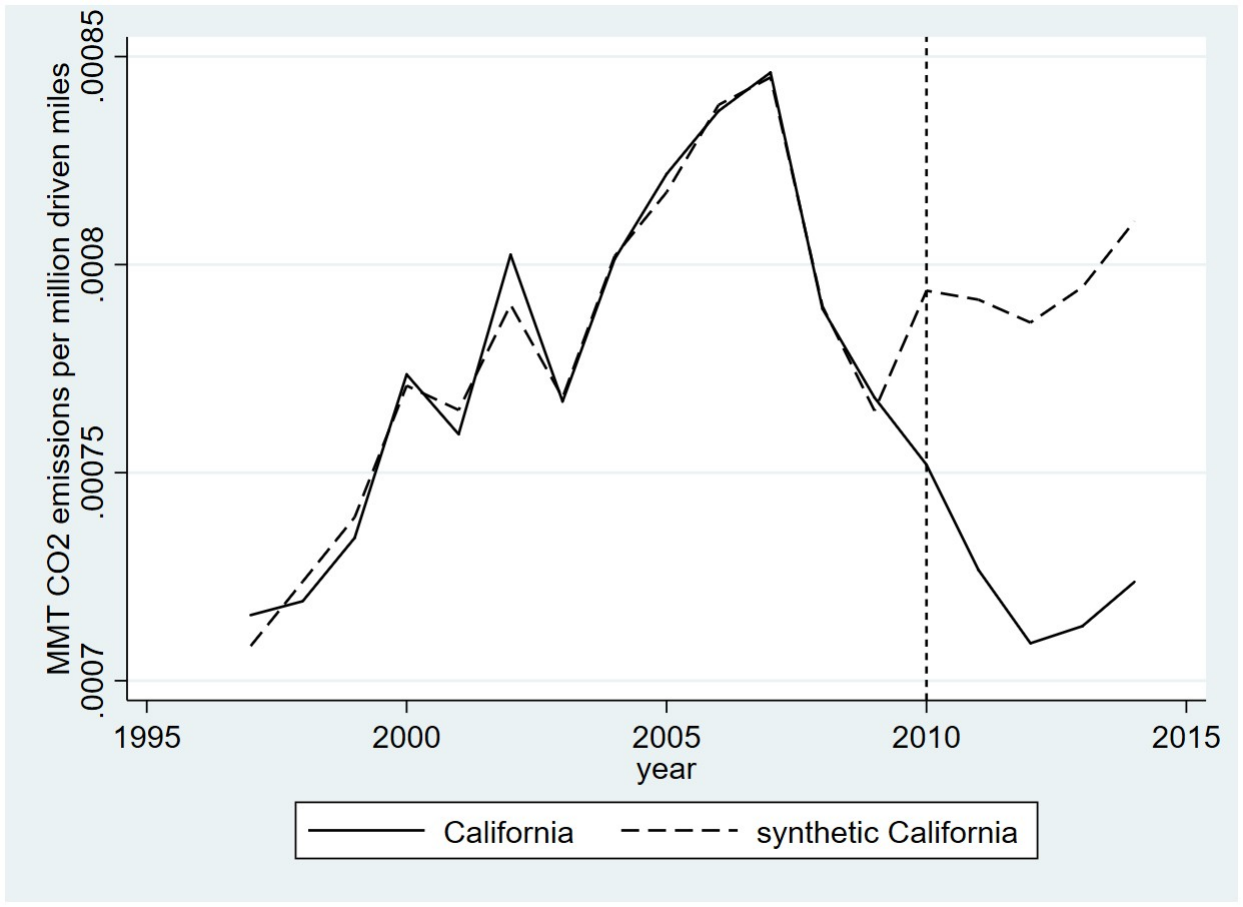
Results of the synthetic control estimation to measure the effect of the LCFS on emissions.

**Fig 3:**
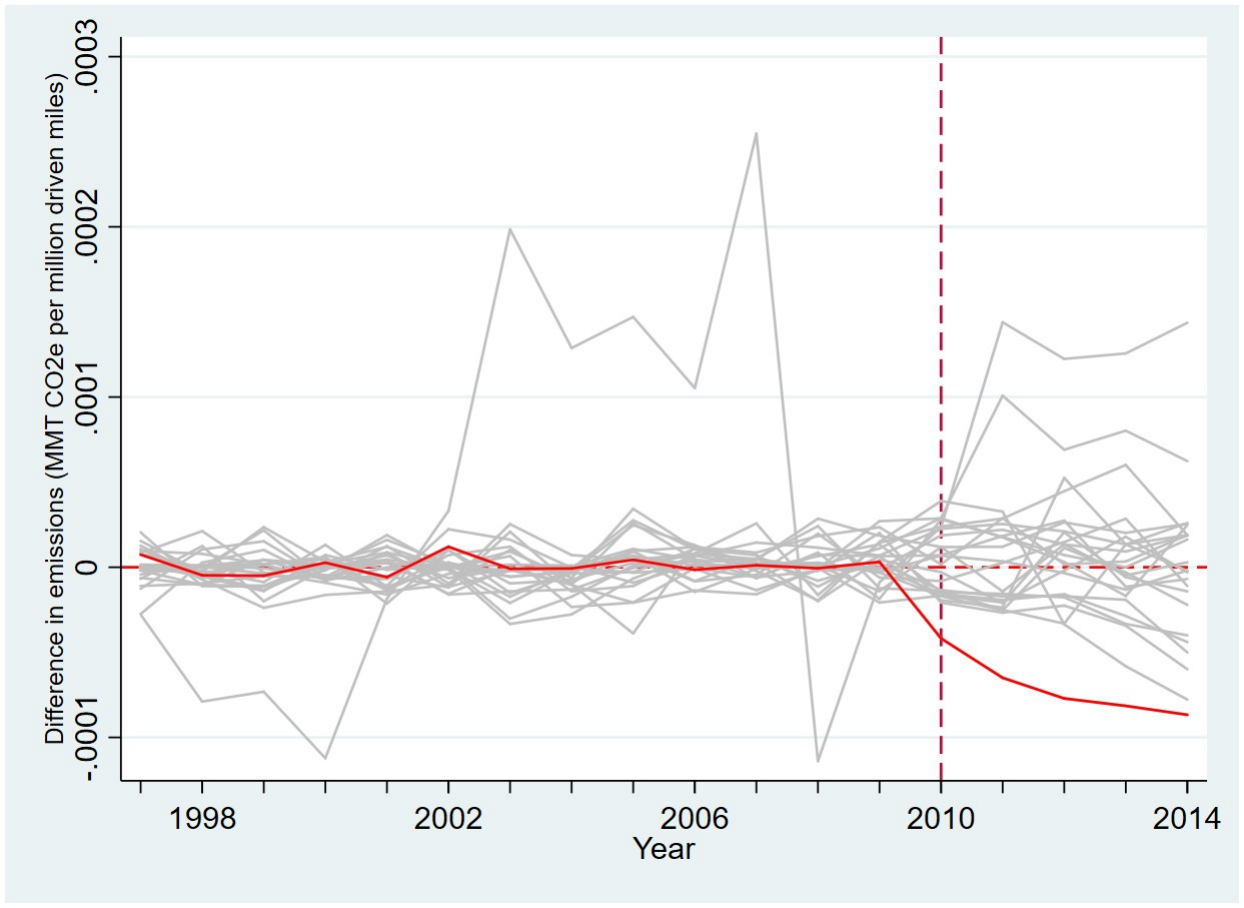
Placebo test of the synthetic control estimation to measure the effect of the LCFS on emissions.

**Fig 4:**
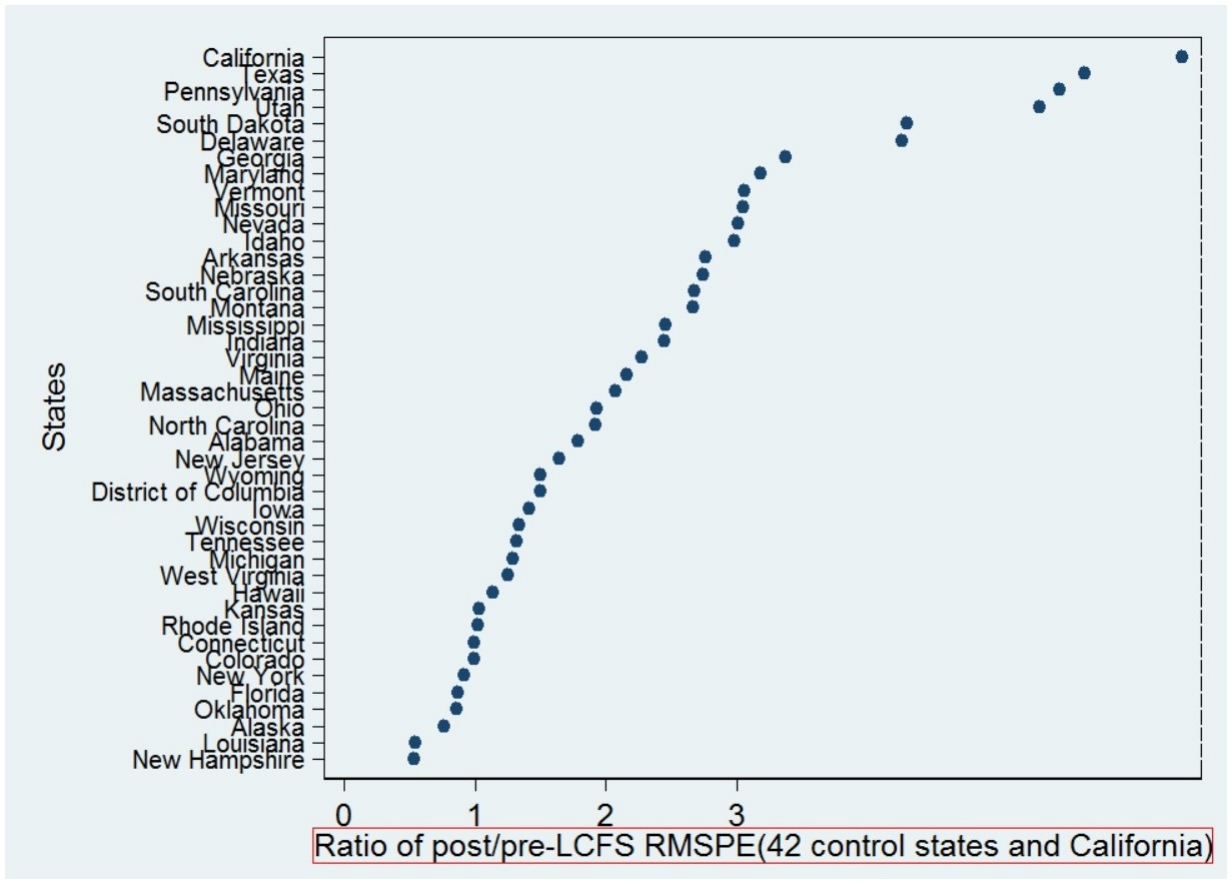
The post-pre RMSPE test of the synthetic control estimation to measure the effect of the LCFS on emissions.

**Fig 5:**
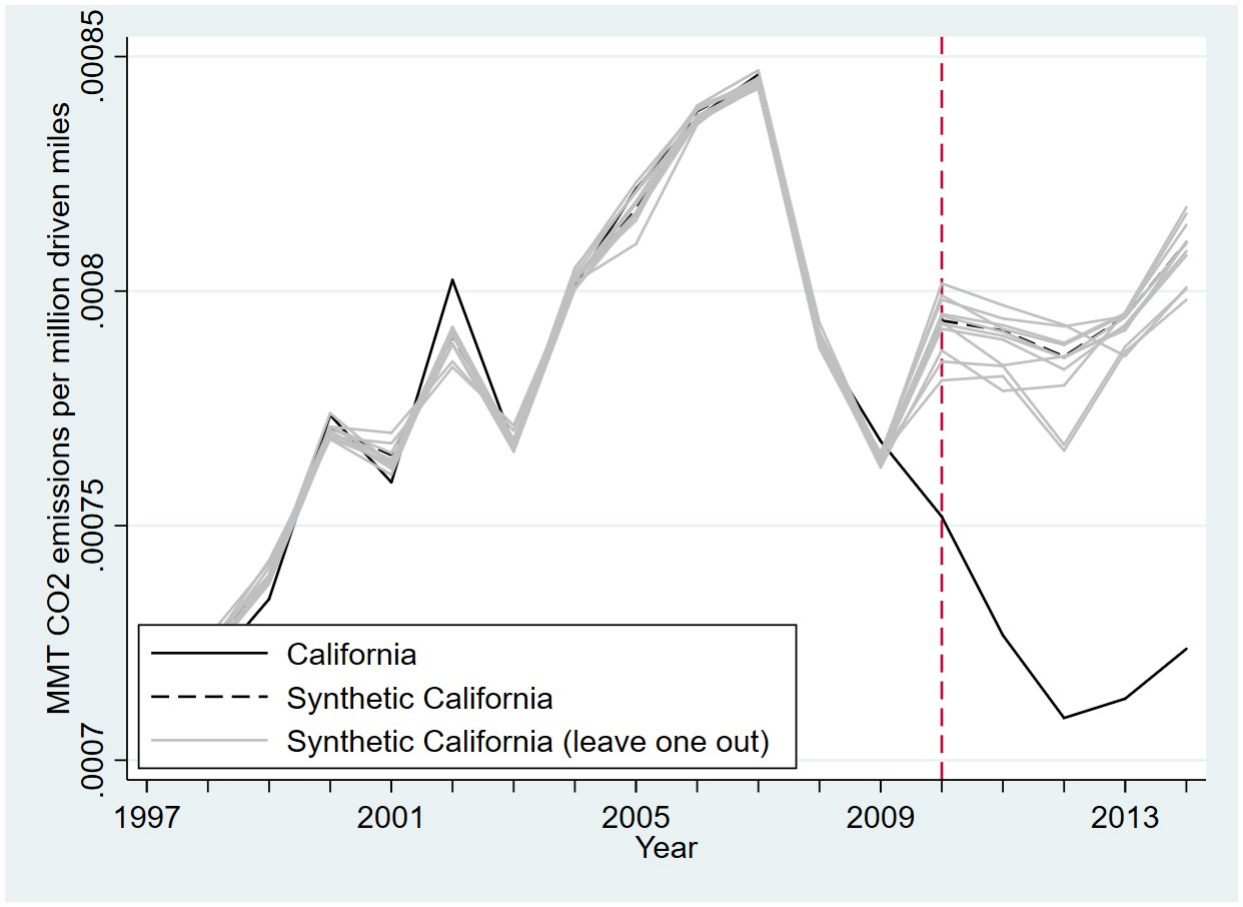
Leave-one-out test of the synthetic control estimation to measure the effect of the LCFS on emissions.

**Fig 6:**
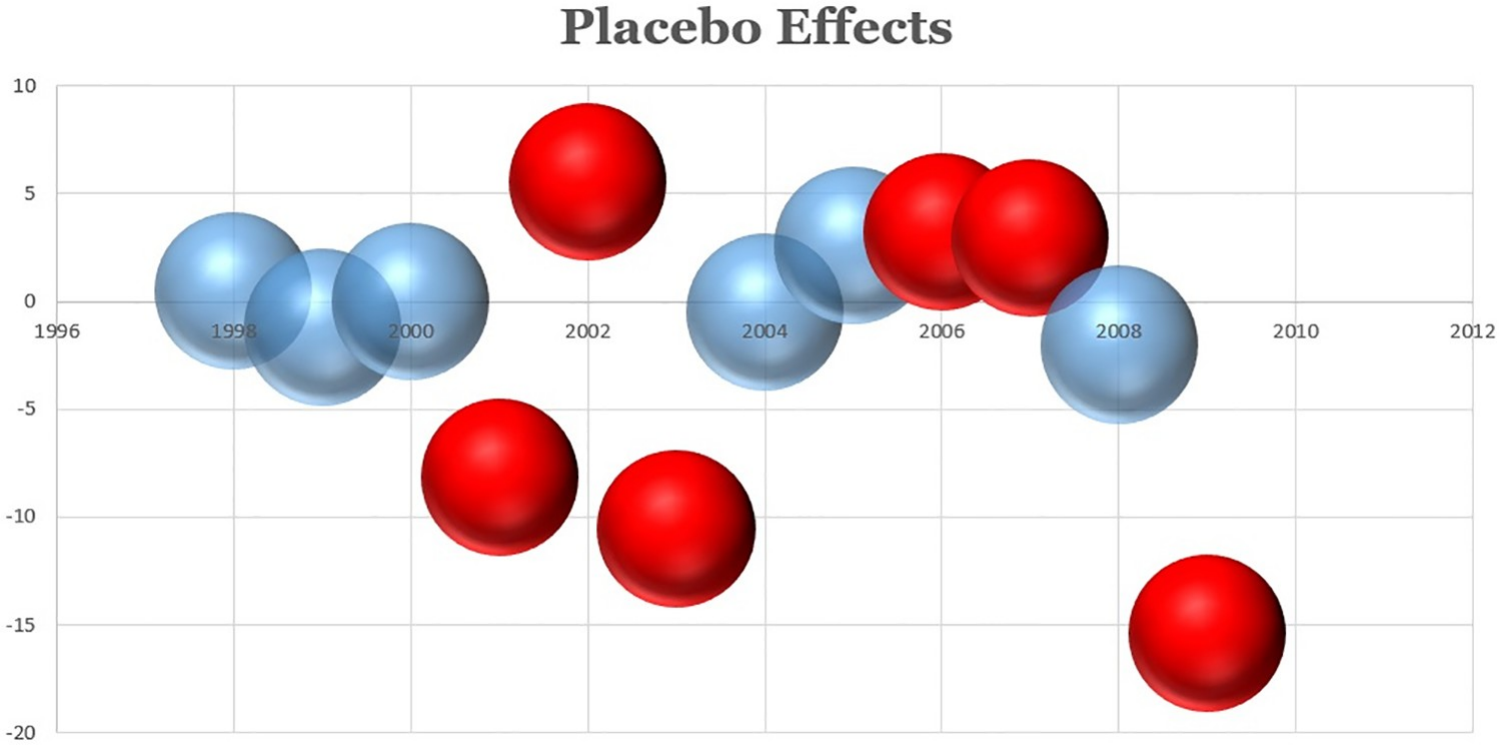
Placebo test for the DID estimation to measure the effect of the LCFS on emissions.
