# Supplementary material for: Does California’s Low Carbon Fuel Standards reduce carbon dioxide emissions?
Source: PLoS One. 2018 Sep 17;13(9):e0203167. doi: 10.1371/journal.pone.0203167 (PMC6141099; doi:10.1371/journal.pone.0203167)
Supplement: S1 Appendix — (PDF) [file pone.0203167.s001.pdf]

S1 Appendix

As discussed previously, in this section we assess whether there is a contaminating effect by the CAT. To test for this possibility, we conduct the SCM analysis for *per capita* CO_2 emissions from the entire industrial sector rather than only the transportation sector. Industry level emissions data are an appropriate measure to analyze the CAT since the CAT targeted the industry as a whole. Figure A shows the *per capita* CO_2 emissions for California and synthetic California. The emission level increased in California compared to synthetic California in 2013, but the effect appears to be small. This analysis provides evidence that CAT does not contaminate our primary results.

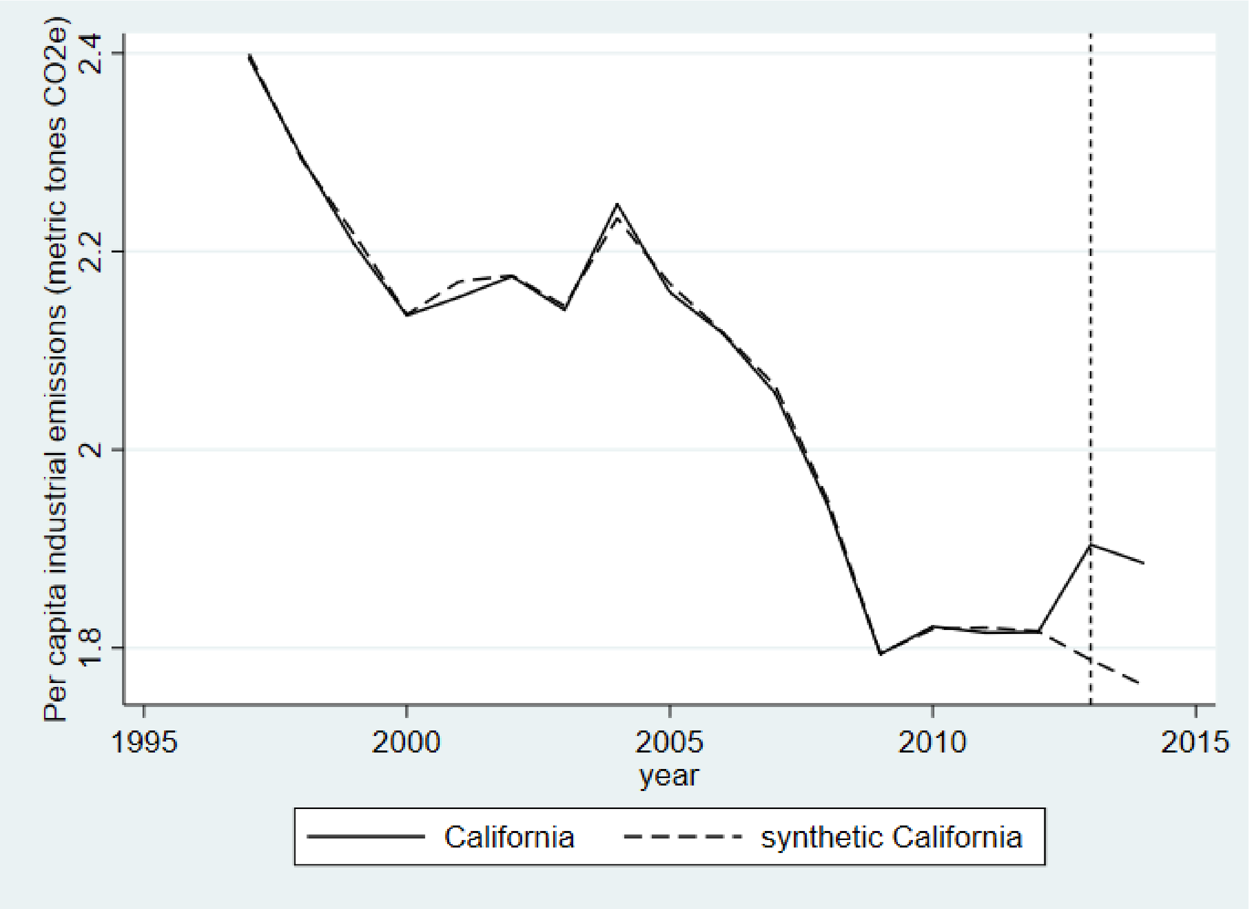

Figure A
