# Supplementary material for: Does California’s Low Carbon Fuel Standards reduce carbon dioxide emissions?
Source: PLoS One. 2018 Sep 17;13(9):e0203167. doi: 10.1371/journal.pone.0203167 (PMC6141099; doi:10.1371/journal.pone.0203167)
Supplement: S2 Appendix — (PDF) [file pone.0203167.s002.pdf]

S2 Appendix

As mentioned in the Identification Strategy section, some state-specific factors related to consumer preferences can contaminate our results. For instance, Plug-in Hybrid Vehicles (PHEV) have the potential to reduce gasoline usage through efficiency gains and eventually decrease total carbon dioxide emissions [47]. [47] study a sample of 877 new PHEV buyers, as well as their driving behavior and find that, based on current California energy scenarios, PHEVs can reduce marginal emissions by more than one-third. Moreover, [48] survey 1000 U.S. residents and show that respondents, who self-identify their political affiliation as "Far Left", are 8.2 times more likely to consider adopting the PHEV technology. However, [48] also find that even respondents with the highest preference for this technology are not willing to pay more than a few thousand U.S. dollars for the technology. Thus, it is not surprising that the market penetration of fuel-efficient and electric cars did not meet expectations. For example, PEV sales reached around 53000 in 2012 and constituted only 0.3 % of vehicle sales in the United States [49]. Moreover, the initial expectation was one million vehicles by 2015 [49].

Controlling the composition of transportation fleet by explicitly documenting the number of fuel-efficient and hybrid vehicles can assure that our results have not been contaminated. However, we study the 1997-2014 period, and in the pre-treatment period, we observe very small variation in the number of fuel-efficient and hybrid vehicles. Thus, we cannot train our model properly, even we control this factor explicitly in the SC estimations. Therefore, we control state-level *per capita* values of *gasoline consumption (barrels)*, and CO_2 emissions in the electricity production (MMTCO2e). *Gasoline consumption* should manifest how much gasoline was displaced due to fuel efficient and electric vehicles. Moreover, *emissions in the electricity production* helps us to control the trend in CO_2 emissions in the electricity production.

On the other hand, policy design has always been a part of political agendas; thus, the political majority of states is an important factor. If California had an increasing inclination towards more environmentally friendly regulations in the pre-treatment period because of various reasons, including political affiliation, synthetic California should reflect those peculiarities. Therefore, we also control the percentage of Democratic Party seats in state legislatures. Additionally, we control state-level *gas taxes (cents/gallon)* and *per capita* values of CO_2 emissions in residential areas (MMTCO2e), *per capita* number of vehicles, *per capita* GDP (USD) and *per capita* road length (miles) in line with the main SCM estimation. Controlling emissions in residential areas gives us another opportunity to capture potential unique patterns in the behavior of California residents.

Figure A shows our SCM results with additional control variables. Synthetic California was constructed with the following weights: Alabama (13.3%), Arkansas (7.1%), Delaware (3.5%),

Florida (23.8%), Louisiana (1.2%), Montana (7.9 %), Oklahoma (7.6 %), Rhode Island (3.4 %), South Dakota (5.6 %), Texas (18.9 %), Utah (4.2 %), and Virginia (3.6 %). We conclude that our main results still hold even when considering the possibility of preference shifts to hybrid and electric vehicles, and the potential impact of political processes.

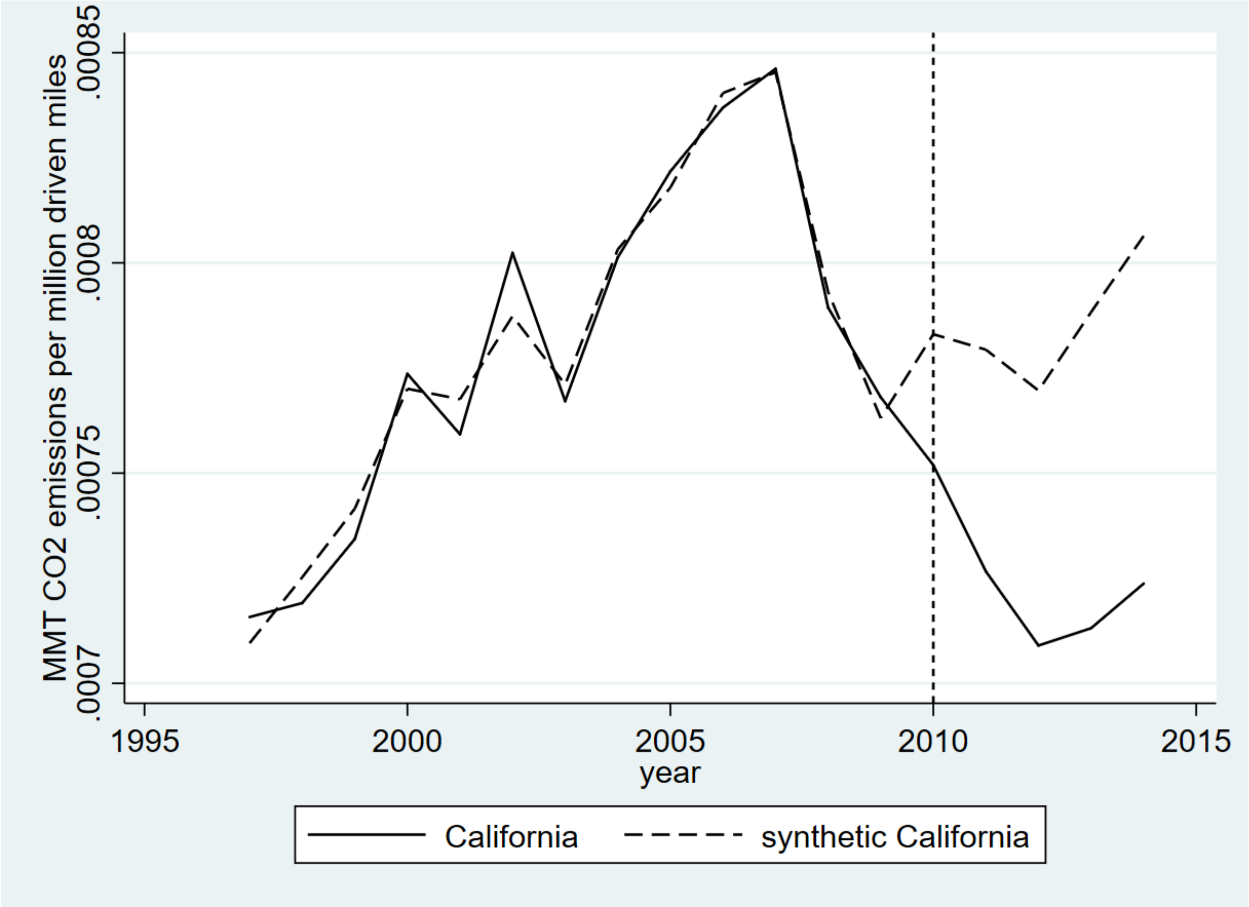

Figure A
