# Supplementary material for: Does California’s Low Carbon Fuel Standards reduce carbon dioxide emissions?
Source: PLoS One. 2018 Sep 17;13(9):e0203167. doi: 10.1371/journal.pone.0203167 (PMC6141099; doi:10.1371/journal.pone.0203167)
Supplement: S3 Appendix — (PDF) [file pone.0203167.s003.pdf]

S3 Appendix

In this section, we resume our discussion about the difference between predicted and actual values for the control variables in the SCM estimations. We drop the control variables with large differences in their estimates for California and Synthetic California. After keeping the selected predicted control variables, we estimate the SCM again and Figure A shows that we still obtain almost a perfect fit between synthetic California and California in the pre-intervention period. If we compare the direction of the curves in the post-intervention period with the results in Fig 2, we can confirm that they are aligned. We conclude that dropping inaccurate predictors does not harm or improve our primary SCM results. Nevertheless, we keep the variables as our control variables to maintain the comparability of the results from the SCM with the DID and Lasso estimations.

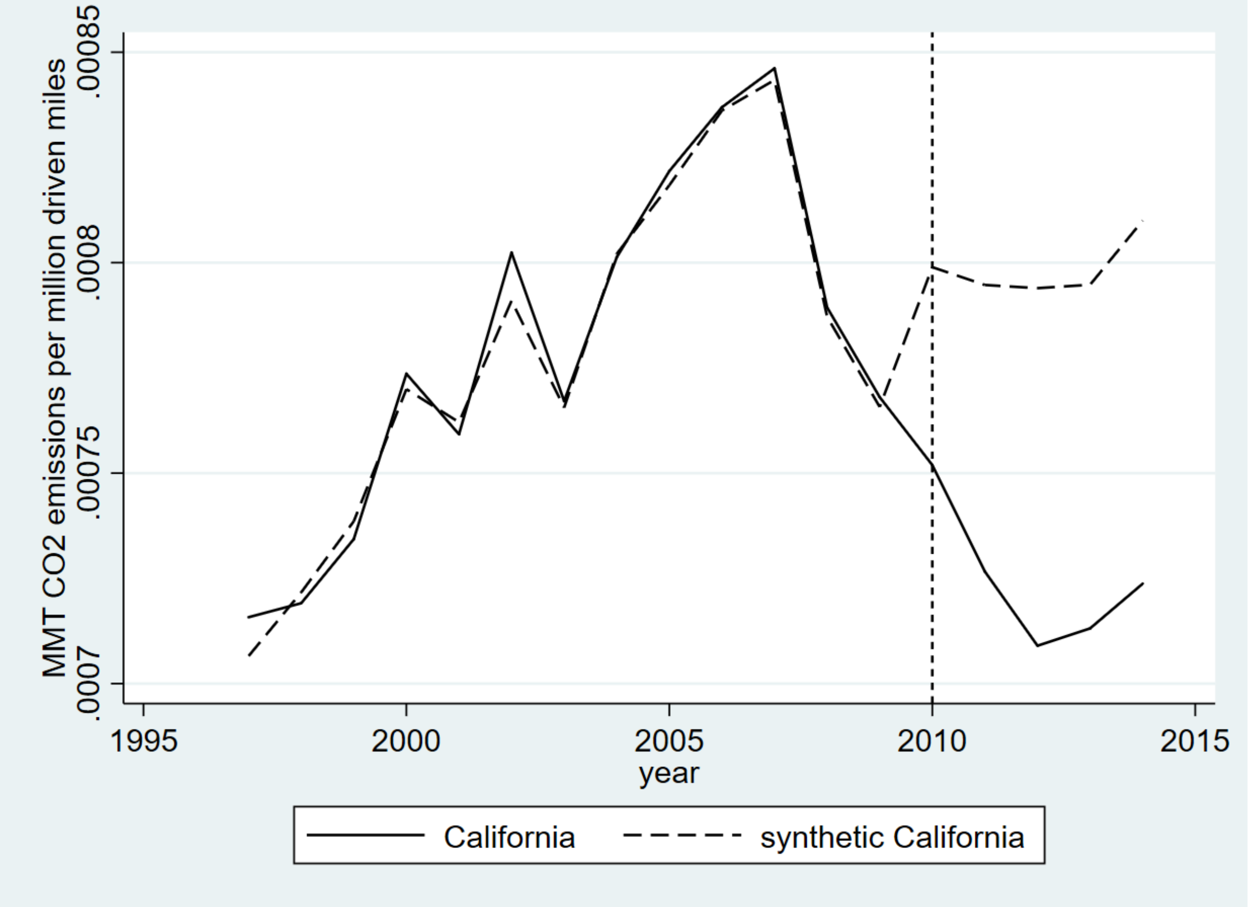

Figure A
